# Towards ‘Fourth Paradigm’ Spectral Sensing

**DOI:** 10.3390/s22062377

**Published:** 2022-03-19

**Authors:** Forrest Simon Webler, Manuel Spitschan, Marilyne Andersen

**Affiliations:** 1 Laboratory of Integrated Performance in Design (LIPID), School of Architecture, Civil and Environmental Engineering (ENAC), École Polytechnique Fédérale de Lausanne (EPFL), 1015 Lausanne, Switzerland; marilyne.andersen@epfl.ch; 2 Translational Sensory & Circadian Neuroscience, Max Planck Institute for Biological Cybernetics, 72076 Tübingen, Germany; manuel.spitschan@tum.de; 3 TUM Department of Sport and Health Sciences (TUM SG), Technical University of Munich, 80333 Munich, Germany; 4 TUM Institute for Advanced Study (TUM-IAS), Technical University of Munich, 85748 Garching, Germany

**Keywords:** reconstruction, symmetric non-negative matrix factorization, nonlinear dimensionality reduction, sparse sensor placement, spectral sensing

## Abstract

Reconstruction algorithms are at the forefront of accessible and compact data collection. In this paper, we present a novel reconstruction algorithm, SpecRA, that adapts based on the relative rarity of a signal compared to previous observations. We leverage a data-driven approach to learn optimal encoder-array sensitivities for a novel filter-array spectrometer. By taking advantage of the regularities mined from diverse online repositories, we are able to exploit low-dimensional patterns for improved spectral reconstruction from as few as p=2 channels. Furthermore, the performance of SpecRA is largely independent of signal complexity. Our results illustrate the superiority of our method over conventional approaches and provide a framework towards “fourth paradigm” spectral sensing. We hope that this work can help reduce the size, weight and cost constraints of future spectrometers for specific spectral monitoring tasks in applied contexts such as in remote sensing, healthcare, and quality control.

## 1. Introduction

Natural signals are compressible functions that represent changes in the spectrotemporal dynamics of physical phenomena [1]. Common examples of natural signals include light and sound. The information contained within a signal is encoded when it is received by an observer. Observers can be biological, such as the human eye, or mechanical (e.g., a digital camera). The most useful “observers” encode signal information into a format that can be read, copied, and shared with others via a process called quantization. Contemporary scientific discovery is increasingly dependent on encoding hardware as numerous autonomous processes require vast amounts of data. In what many are now calling the “fourth paradigm in scientific discovery” [2], efficient data transcoding is paramount. Herein, we approach the metrological process from the perspective of information science starting with quantization and concluding with reconstruction. We hypothesize that by exploiting regularities in existing datasets, we can develop optimized non-uniform protocols for spectral sensor placement and use adaptive methods to maximize reconstruction efficiency. While we focus on spectral sensing for visible light, the methods discussed are applicable to any set of signals characterized by locality and compositionality.

### 1.1. What Is “Spectral” Sensing?

Spectral sensing is used in a number of applied contexts including healthcare, remote sensing, and quality control [3,4,5]. To meet the requirements of this highly diverse field, current compact spectral imaging solutions include systems based on the following technologies:

1. Multispectral, filter-array sensors;
2. Compressive spectral sensors;
3. Lens-encoders;
4. Spectral super-resolution;

While each application of spectral sensing has different requirements for what is considered “spectral” resolution, we can broadly identify three main classes: (1) tristimulus; (2) multispectral; and (3) hyperspectral imaging. Pixel-based, red, green, blue (RGB) is perhaps the most ubiquitous “spectral” signal and is used for color rendering and other imaging purposes. While RGB has historically been excluded from spectral sensing technologies, interest in recovering broadband spectral signals from RGB has increased over the years [6,7,8,9,10,11]. So-called “RGB-to-spectrum” approaches are, however, limited in their applicability to broadband (low-complexity) signals and generally fail for signals with low autocorrelation. Multispectral imaging is an exciting emerging field and typically refers to systems capturing between 3 and 10 channels with bandwidths greater than 20 nm [12]. Common applications are in color evaluation for quality control and remote sensing [13]. Advances in optical filters and semiconductor technology have also improved the size, weight, power, and cost constraints, making hyperspectral sensing a more attractive middle ground [14]. Hyperspectral imaging is perhaps the most diverse class reserved for devices measuring more than 10 channels [15]. For imaging applications, hyperspectral cameras can have between 512 and 2048 channels over the visible domain making them extraordinarily information-rich. While devices in each of these classes all collect “spectral” data, the range in resolution covers three orders of magnitude.

### 1.2. Existing Methods

Many compact spectral sensing methods exist based on the aforementioned technologies. Recent interest in multi-spectral filter-array technology has, in particular, attracted a lot of interest. Methods of multispectral demosaicking have improved low-cost “single shot” imaging [16] as well as compressive methods [17]. Alternative approaches look for statistical regularities to exploit for task-specific applications. A plethora of work in natural image statistics has motivated approaches based on scene optimization [18] and RGB images [8,19,20]. Increasingly, however, focus has shifted towards data-driven computational methods to recover as much information as possible from low-fidelity measurements. This includes improving the optimization frameworks for diffractive achromats (DAs) [21] and constructive improvements for the alternating direction method of multipliers (ADMM) optimization in solving ill-posed reconstruction problems [22].

Contemporary approaches to compressive spectral sensing rely on a diversity of sensor technologies from liquid crystal phase retarders [23,24,25] to stacked array spectrometers with broadband filters [26]. Emergent technologies such as quantum dot and nanowire spectrometers [27,28] also show great potential to disrupt the spectral sensing space. At the same time, the manufacturing cost of “single shot” compact filter-array spectrometers with 3–20 channels and bandwidths in the 20 nm range have dramatically decreased to a fraction of the cost of conventional scanning spectrometers [29,30]. Regardless of the underlying technology, each measurement device maps data from the “natural” dimension to a hardcoded “measurement dimension” defined by the resolution of the device. Towards this end, we are interested in applying a data-driven approach to investigate the theoretical boundaries constraining reconstruction performance in the future generation of compact sensing hardware.

### 1.3. Problem Statement

While determining the threshold of what constitutes “spectral sensing” is highly contextual, advances in applied math and signal processing have demonstrated that natural signals exhibit a high-degree of redundancy. Formally, this means that most natural signals are encoded in a smaller dimension *below* the theoretical limits set by the Shannon–Nyquist sampling theorem [31]. As we are interested in investigating the magnitude of information required to reconstruct a spectral power distribution from low-dimensional data, we will try and find the best low-rank representation (i.e., *embedding*) given a set of prior observations. Determining a theoretical limit and workable latent space for visible spectra will not only help to improve how spectral data are measured, compressed, and stored, but also in how they are classified.

Efficient sensing is a two-step process requiring the informed design of an optimized encoder $g_{\phi }$ and an adaptive reconstruction algorithm characterized by $f_{\theta }$. Thus, the contributions herein are two-fold. First, to determine the optimal sensor locations for the reconstruction of broadband (low-complexity) and narrowband (high-complexity) spectra. Second, to develop an a data-driven reconstruction algorithm (SpecRA) that balances simplicity and reconstruction fidelity. Together, we show that a combined workflow can account for real-world engineering and fabrication constraints on spectral sensors to determine *what are the maximally informative dimensions of visible spectra* in theory and practice.

## 2. Beating Nyquist

In 2005, our understanding of the theoretical limits of sampling rate for bandlimited signals were such that if a signal is sampled at a frequency *f*, perfect reconstruction is only guaranteed if the bandwidth b<f/2 [32]. This observation is a result of the Shannon–Nyquist sampling theorem and while this theorem still holds today, two seismic advances in applied math have forced engineers to contextualize it in a new light. First, in 2006, it was demonstrated that sub-Nyquist sampling was possible without violating the theorem by requiring the signals to be *sparse* (i.e., compressible) in a generic basis [31,33]. This approach is called *compressive sensing* and has since revolutionized data collection by initializing compression at the point of quantization. Second, the increase in data availability has made it possible to feature mine prior observations for statistical regularities. These regularities can then be used to exploit symmetries and other structural properties in order to make inferences that further maximize reconstruction performance. In this way, the “datafication” of our world has imparted huge implications for metrology in general and spectral sensing in particular. In this paper, we will specifically show how advanced domain knowledge can be used to optimize the measurement process.

## 3. What Is Reconstruction?

Simply defined, *reconstruction* refers to a process of recovering a signal from a set of limited measurements [1]. In practice, this can be done several ways depending on the application and in context, all of which result in solving the following problem:

(1) $$\min\;K\left(\hat{s},\;s\right)\;\;\;\;\;\;\;\;\;\;\;\;\mathrm{subject}\;\mathrm{to}\;\;\;\;\;\;\;\;\;\;\;\;\hat{s}=f_{\theta }\left(y\right)$$

**Figure 1:**
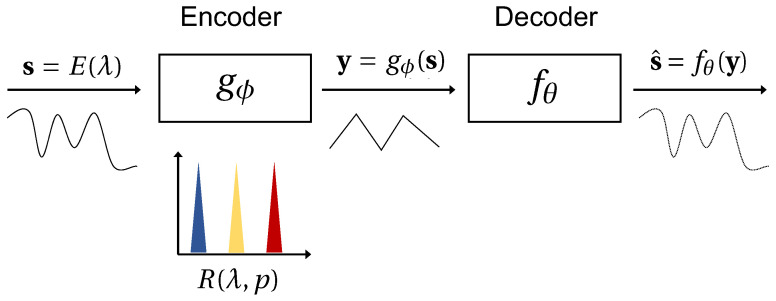
Spectrometry is the process of encoding spectral data to a measured dimension *p* from the infinite natural signal dimension. This process is visualized in the above graphic and mathematically written as $g_{\phi }:R^{\infty }\to R^{p}$. The measured data can then be reconstructed to a target high resolution n>p such that $f_{\theta }:R^{p}\to R^{n}$. Here, the encoder $g_{\phi }$ is a filter-array spectrometer with some transmission functions R(λ,i) for i=(1,…,p) and $f_{\theta }$ represents a reconstruction algorithm. This structure is analogous to that of an autoencoder where the learned encoder weights would replace the response functions of the physical spectrometer and *p* is the dimension of the hidden layer.

Here, $y=g_{\phi }\left(s\right)$ is a measurement of a high-dimensional signal s=E(λ) by an encoder $g_{\phi }$ reconstructed by a function $f_{\theta }$ (c.f., Figure 1). The function K being minimized can be any metric or norm capturing the dissimilarity between the reconstruction and the ground truth. If the signal is sampled at or above the Nyquist rate, reconstruction can be as simple as constructing a linear fit through the sub-sampled points (we call this the “interpolation regime”). Alternatively, if the signal is undersampled but known to exhibit unique statistical regularities, “reconstruction” can also be a process of finding a match among known prior observations. In both examples, we say the reconstruction is “naïve” in the sense that interpolation and pattern matching are inherently trivial tasks.

If the reconstruction is *lossy*, performance metrics are accompanied with a compression power score to contextualize the trade-off between complexity and descriptivity. The compression power is defined as the ratio between the uncompressed and compressed file size (e.g., defined by the vector length). What can be misleading about the compression power is that in the limit that the compressed file size obtains information saturation (i.e., is sampled at a lossless rate via Nyquist or CS) the compression ratio becomes meaningless when the uncompressed signal dimension is increased. This is because the features used for lossless reconstruction are fully formed at a given resolution, and artificially increasing the resolution of the uncompressed file will inflate the compression power. Towards this end, we propose a slightly amended performance metric that encapsulates the about of “work” performed by the algorithm $f_{\theta }:R^{p}\to R^{n}$ in order to reach a target error depending on the available information:

(2) $$W=\max[p^{-1}n\left(\Delta -\max\left[\gamma ,\mu \right]\right),\;0]$$

where Δ is the target performance threshold, γ is the percent of signals matching signals in the Kanji, and μ is the percentage of signals recovered at or above the Nyquist rate. We can think of *W* as the penalized compression power that is set equal to 0 when the reconstruction algorithm fails to outperform either the matching problem or interpolation. Given that we reconstruct a set of observations encoded by $g_{\phi }$, from a Kanji $K\in R^{m}$, we can define the proportion of adequately reconstructed signals as the ratio of the cardinalities of *S* and **S** where $S=\{\;s\;|\;s\in S\;\wedge \;K\left(s,\;\hat{s}\right)<\epsilon \;\;\forall \;\;\hat{s}=f_{\theta }\left(y\right)\;\}$ is the subset of reconstructed observations adhering to a dissimilarity score less than ϵ defined by a function K appropriate for the underlying datatype. For spectral sensing applications, this is typically the spectral angle (SA) or another derivative index corresponding to the spectral information divergence [34,35].

When we say that the algorithm has to “work”, what we mean is that unlike in pattern matching and interpolation tasks where there is no optimization procedure taking place, reconstruction has to “add” information that is not trivially available. When signals have no corresponding match and are sampled in the sub-Nyquist regime, the reconstruction problem formulated in Equation (1) becomes:

(3) $${\min\;∥x∥}_{1}\;\;\;\;\;\;\;\;\;\;\;\;\mathrm{subject}\;\mathrm{to}\;\;\;\;\;\;\;\;\;\;\;\;\hat{s}=\sum_{i=1}^{m}x_{i}\;k_{i}$$

where **x** is a coefficient vector and $k_{i}\in K$ is a prior observation contained in our “Kanji” (a Pareto-optimal library distinct from a learned dictionary). A fundamental property of signal reconstruction is that the more complex a signal is, the more basis modes are needed to accurately approximate that signal. One of the consequences of deriving **K** from a big dataset **L** is that the likelihood that a “new” observation has already been measured and exists in an accessible dataset is high. Consequently, reconstruction in the era of big data is less about adhering to a specific methodology and instead about finding the most efficient process required to return the true signal from an encoded measurement. Towards this end, ensuring that **K** comprises real spectral observations ensures that the “missing” information in an undersampled measurement can be “filled in”. That said, when we take a measurement **y**, the only information we have is that of the encoded signal. This means that we have to “trick” the algorithm by solving:

(4) $$\min\;∥x^{\prime }{∥}_{1}\;\;\;\;\;\;\;\;\;\;\;\;\mathrm{subject}\;\mathrm{to}\;\;\;\;\;\;\;\;\;\;\;\;\hat{y}=\sum_{i=1}^{m}x_{i}^{\prime }\;g_{\phi }\left(k_{i}\right)$$

with the assumption that $x^{\prime }\approx x$. Given that **K** is not a generic basis, the guarantees of compressed sensing do not hold in this case. Instead, the efficacy of this assumption is constrained by the ability of signals in **K** to preserve a unique structure in the measured dimension.

**Figure 2:**
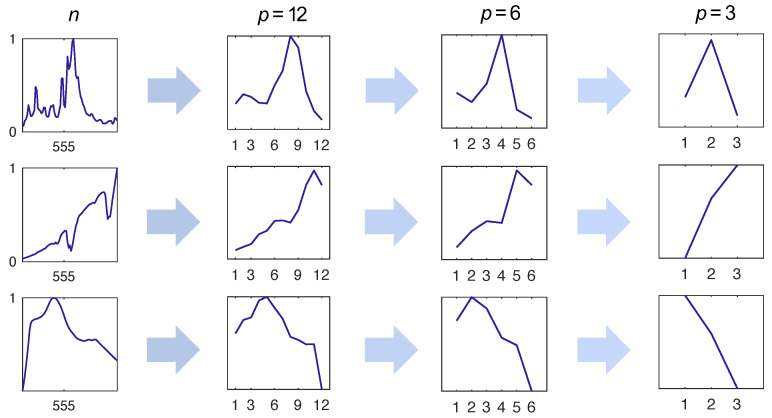
Unique identifying features of the signal are lost when the resolution of the sensor is decreased. Here, we show the effect of uniform sampling. Preserving unique features in the measured dimension is important for ensuring agreement between $x^{\prime }$ and **x**.

Of course, not all measured dimensions are the same. For example, we show in Figure 2 what happens when signals are sampled with a uniform encoder. A central aim of this work is to investigate other sampling protocols by constructing $g_{\phi }$ from bases, learned via different decomposition methods. We hypothesize that non-uniform sampling procedures will be informed by the features shared by many real-world spectra and will therefore result in a greater preservation of “uniqueness” in the measured dimension. Furthermore, by extension, we hypothesize that preserving structure in the encoded output will result in greater reconstruction performance for undersampled data.

Looking at Figure 2, one may also question why we would bother forming the reconstruction problem as Equation (4) in lieu of training a neural network on **K**, thereby learning a map between the measured and target dimensions. We refrain from taking such an approach for two reasons: first, it is well known that “black box” models do not generalize well and this has, in fact, been demonstrated to be the case in the application of recovering visible spectral distributions from encoder-array spectrometers [36,37]; second, we lose any interpretability or contextual reference of the scene. While Equation (4) may seem trivially simple, enforcing sparsity via the $l_{1}$-norm has demonstrated incredible success in not only reconstructing low-dimensional measurements, but also in reconstructing them in a way that mimics the underlying physical system [38]. This observation has led some to claim that parsimony is the ultimate physics regularizer [39].

## 4. Towards Data-Driven Bases for Reconstruction

**Figure 3:**
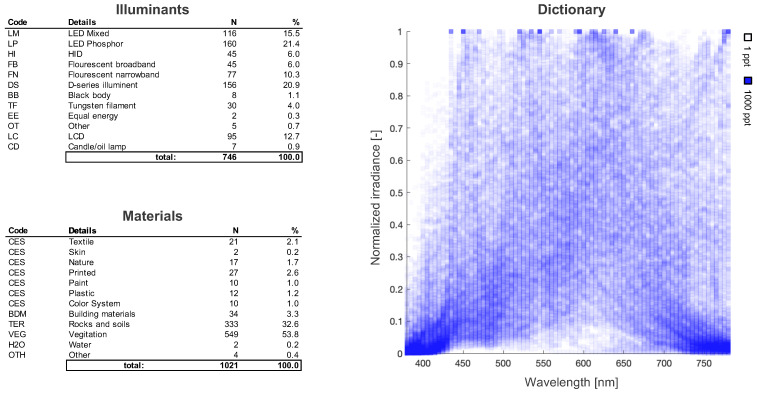
A high-level description of the contents of our spectral libraries for the illuminants and material reflectances. Of the 746 illuminant spectra, 146 are theoretical and 600 are real. The 1021 reflectances correspond to material samples under equal energy illumination. We can see that despite the variety of illuminants and materials, there are clear regularities in signal space (e.g., very few spectra have high relative power between 380 and 430 nm).

Variation in spectral distributions is created from interactions between light and matter. In order to derive a basis, we first need to compile some observable data. Here, we compile a library of illuminant and reflectance spectra from available open source datasets [40,41,42,43]. A high-level description of our library **L** is summarized in Figure 3. Since each spectrum was sampled at different frequencies, we normalized all spectra within the visible range from 380 to 780 nm and re-sampled them using standard interpolation methods. Within **L**, we have 401 spectra which were used as a representative set for color rendition studies in addition to 99 color evaluation samples (CESs) uniformly distributed within the natural color system (NCS) gamut [44,45].

Additions to the illuminant class include mobile and computer screen as well as high-intensity discharge (HID) sources used in street lighting, and daylight spectra for various sun angles above the horizon in urban and rural settings [46]. We acknowledge that this set is not complete; however, we believe that from a mechanistic perspective, the space of available spectral illuminants is sufficiently sampled and is likely overcomplete for some sub-classes [47]. Like pixel space, the vastness of signal space means that natural signals are inherently rare with the vast majority of signals containing no information [48]. Even if future spectral measurements are naturally sparse in **L**, there is a lot of redundancy making **L** computationally heavy. This is where our learned Kanji can be useful. If **K** comprises the same features as **L**, then we can use **K** to derive a low-rank basis (with the default being uniform placement). Towards this end, we investigate the following sparse coding methods:

1. Singular value decomposition (SVD);
2. Symmetric non-negative matrix factorization (SymNMF);
3. Sparse dictionary learning (SDL);
4. Deep autoencoders (DAE).

### 4.1. Singular Value Decomposition

The simplest data-driven basis can be derived by computing the first *r* column vectors of the unitary matrix of the singular value decomposition [39]. Given some representative data **K**, a basis $\Psi_{r}$ can be found by solving:

(5) $$K=U\Sigma V^{*}\;\;\;\;\;\;\;\;\;\;\;\mathrm{where}\;\;\;\;\;\;\;\;\;\;\;\Psi_{r}=U_{r}\Sigma_{r}.$$

The main advantage of SVD is that it can be quickly and efficiently performed in most computational software packages. The resulting basis (columns of $\Psi_{r}$) are ordered with respect to the strength of their contribution in representing variance in the original data matrix. When plotted, it is evident that any similarity to real-world spectral power distributions is lost. Instead, we can think of the basis vectors as defining an abstract “feature space”. While the advantage of an SVD-derived basis is simplicity, the drawback is that the bases can only be used to find a linear map (i.e., a relatively simple relationship given the abilities of modern deep autoencoders). In fact, we can think of SVD as a special case of a DAE wherein the encoder weights describe a linear relationship.

### 4.2. Symmetric Non-Negative Matrix Factorization

While the SVD basis works well capturing the features of **K**, it notoriously lacks interpretability when applied to physical systems where negative values may be meaningless. Within the context of designing a spectral imaging sensor, the response sensitivities of each channel must be positive because there is no physical way to interpret negative sensitivity. Towards this end, we implement *symmetric* non-negative matrix factorization (SymNMF) [49,50]. SymNMF overcomes the pitfalls of other algorithms insofar as it is capable of capturing *nonlinear* cluster structures (unlike standard “brother” non-negative matrix factorization). Even more interestingly, SymNMF optimization is independent of the eigenspace of the affinity matrix (unlike spectral clustering). Furthermore, the affinity matrix **A** can be defined with respect to any appropriate distance metric given a priori knowledge of the datatype. The minimization problem for SymNMF is defined as

(6) $$L\left(\Psi ,r\right)=\underset{\Psi \geq 0}{\mathrm{argmin}}{∥A-\Psi \Psi^{\prime }∥}_{F}^{2}$$

where *r* is the rank of Ψ. If the number of spectra in **L** is *n*, then **A** is a square n×n matrix where each element in **A** corresponds to a measure of distance between observations. Formally, we define **A** elementwise as

(7) $$a_{i,\;j}=\{\begin{array}{cc} K(k_{i},\;k_{j}) & \mathrm{if}\;i\neq j\\ 0 & \mathrm{if}\;i=j \end{array}$$

where $K(k_{i},\;k_{j})$ is a similarity measure (e.g., Euclidean distance) between $k_{i},\;k_{j}\in$ **K**. Here, $\Psi_{r}$ has *r* columns corresponding to the learned basis vectors. One of the core benefits of SymNMF is that *d* can be selected via knowledge of the underlying datatype. In the most abstract applications, *d* may best be represented by information theoretic measures such as the normalized information and compression distances [51,52].

### 4.3. Sparse Dictionary Learning

Sparse dictionary learning (SDL) is a sub-domain of sparse representation that spans a number of algorithms, most notably: the method of optimal direction (MOD) [53,54]; *k*-singular value decomposition (K-SVD) [55]; and online dictionary learning (ODL) [56], which is commonly implemented for its competitive speed [57]. While it may appear that learned dictionaries are naturally superior, it is important to understand their benefits and shortcomings. First, SDL may be unnecessary if the data are naturally sparse in signal space [39]. Second, dictionary learning algorithms tend to be computationally expensive because they require multiple iterations to converge on the optimal solution. Regardless, their applicability in sparse approximation should not be ignored and while the atoms do not necessarily retain their similarity to real-world spectra, they do share the most similarities than any of the other methods and do not produce negative values if the input data are nonnegative. We seek to find a dictionary $\Psi_{r}$ by minimizing the following loss function reported in [58]:

(8) $$L\left(y,\Psi_{t}\right)=\arg\;\min_{x^{\prime }}∥y-\Psi_{t}x^{\prime }{∥}_{2}+\beta {∥x∥}_{1}$$

Here, β is a sparsity-promoting coefficient. This loss function is commonly referred to as the sparse-coding or LASSO regression [59]. LASSO balances sparsity with model complexity in order to promote a dictionary with low cross-validated error and is one proposed approach to solving the $l_{1}$-minimization problem framed in Equation (3).

### 4.4. Deep Autoencoders

Autoencoders refer to a specific class of artificial neural networks whose aim is to learn the most efficient encoding of some data in a target low-dimensional representation (latent space) [60]. The architecture of an autoencoder is roughly represented by the sketch in Figure 1 where the encoding weights Ψ are learned via the loss function:

(9) $$L\left(y,\Psi \right)=\arg\;\min_{x^{\prime }}\frac{1}{2}{∥\hat{y}-y∥}^{2}+\beta \Omega_{w}+\lambda \Omega_{s}$$

where $\Omega_{w}$ acts as an $l_{2}$ penalty on the encoder weights and $\Omega_{s}$ enforces sparsity via the Kullback–Leibler (KL) divergence [61]. While the loss function for the autoencoder requires more unpacking than others, the key take-away is that the mean squared error (MSE) is minimized between the *learned* representation $\hat{y}=f_{\theta }\left(y\right)$ and **y** for some latent basis Ψ given some regularization constraints on the weights associated to each basis vector, and a sparsity constraint on the reconstruction of the output. In essence, the goals and ambitions are well aligned with the other methods but with an added degree of flexibility. Equation (9) is closely related to the sparse relaxed regularized regression (SR3) method [62] aimed at finding a less restrictive loss function.

### 4.5. Implementation via QU Factorization

To summarize out steps up to this point, we amassed a library $L\in R^{n}$ of available online datasets without processing it in any way. We then used a subset $K\in R^{m}$ as an input to a number of well-known sparse-coding methods to arrive at four candidate low-rank bases $\Psi_{i}\in R^{r}$ for n≫m>r. We now want to use these bases to design different non-uniform encoders by assigning response functions to the pivot points derived via QU factorization [63]:

(10) $$\Psi_{i}P_{i}=QU$$

**Figure 4:**
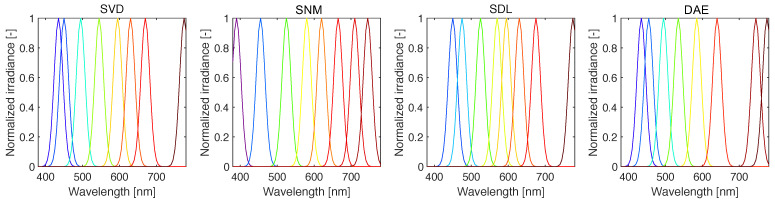
Response functions (shown here for p=8, σ=18 nm) constructed via Equation (11). Here, the peak responsivity corresponds to the pivot points derived via QU factorization.

Here, a pivot matrix **P** is derived for each of the four “data-driven” bases (n.b., the uniform basis is not subscripted). Equation (10) can be solved using preset commands in most computational suites, taking the basis $\Psi_{i}$ as the only input and outputting the pivot points. The resulting pivots (non-zero entries in **P**) correspond to the peak wavelengths used to construct our encoder. From these points, we can define a response function by fitting a Gaussian distribution consistent with most available filters used in array-type spectrometers [14]. The generic structure for a sensor with approximate Gaussian responsibility over a wavelength range λ∈Λ, which is defined as

(11) $$R\left(\lambda ,p\right)=\exp[-\frac{\lambda -\Lambda (P_{ij})}{2\sigma^{2}}]$$

where $\Lambda (P_{ij})$ is the peak wavelength corresponding to the non-zero element of the *p*-th column of **P** (for a total of *p* channels), and σ is the full-width at half-maximum (FWHM). As it is infeasible to manufacture photodiode sensors with single-wavelength sensitivity channels (the diodes themselves are made from semiconductors with limited physical properties), available filters have FWHM values such that σ∈[18,25] nm [14]. In Figure 4, we show example response functions constructed via Equation (11).

An encoder, $g_{\phi }$, with *p* channels, defines a measurement process:

(12) $$g_{\phi }:R^{\infty }\to R^{p}$$

As with all “natural” non-bandlimited signals, $E\left(\lambda \right)\in R^{\infty }$ requires that we make an assumption that $E\left(\lambda \right)\in R^{n}$ where *n* is finite and p≪n. When p<2Klog(n/K)+(7/5)K, where *K* is the sparsity of the coefficient vector [33], then $g_{\phi }$ is a *lossy* compressor as the rate is below the minimums required for lossless reconstruction via Shannon–Nyquist and compressed sensing. We can further minimize information loss by designing $g_{\phi }$ in a way that leverages the domain knowledge that the underlying datatype $g_{\phi }$ will likely encode. To do this, we need to construct a simple mathematical model for $g_{\phi }$ that simulates the measurement process of a real-world physical sensor. This is performed by modeling the output current as proportional to the sum of the response function multiplied by the unknown spectral distribution:

(13) $$y=\epsilon \left(\lambda ,p\right)+\sum_{i=1}^{\lambda_{⊤}}R\left(i,p\right)E\left(i\right)$$

where $\lambda_{⊤}=\max\left(\Lambda \right)$ and ϵ(λ,p) is the per-wavelength measurement error associated with the channels. If we assume the response functions are roughly Gaussian, the relative differences between measured points are only preserved if the response functions are the same width (which is not always the case in real-world sensors).

## 5. Specra: An Adaptive Reconstruction Framework

Reconstruction is broadly characterized by three regimes depending on the accessibility of information: matching, reconstruction and interpolation. When signals are easily describable in **K**, there is little risk of overfitting, but when signals exhibit unseen features, adding a greater number of elements to **K** does not always imply greater reconstruction performance. What we seek to accomplish with SpecRA, is to develop an adaptive framework that uses the knowledge of **K**, together with the measured signal **y**, to triage observations as they arrive at the sensor in order to apply the reconstructive method with the highest probability of success. The core decision making comes down to the value of the following measure, which we refer to as the relative “rarity” of the measurement **y** relative to the encoded elements in the reference set $k_{i}\in K$:

(14) $$R=\frac{1}{m}\sum_{i=1}^{m}K\left(y,\;g_{\phi }\left(k_{i}\right)\right)$$

**Figure 5:**
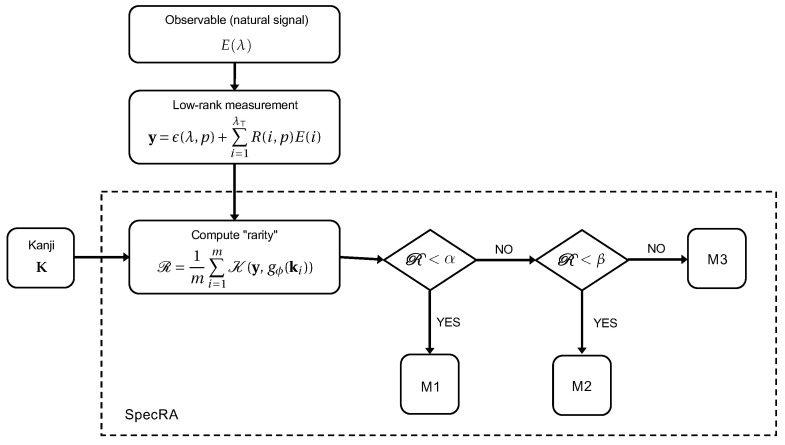
The SpecRA algorithm sorts raw measurements based on their rarity R relative to a Kanji **K** and applies an appropriate minimizing optimization (i.e., M1, M2, or M3). This process helps prevent both underfitting and overfitting, making for an adaptive and more generalizable framework. In general, the greater the R, the greater algorithmic “work” is required.

The challenge with this approach is that measured signals lose much of their unique features during the encoding process. In the previous section we outlined a workflow to improve the preservation of feature structure by deriving data-driven non-uniform sampling alternatives. Here, we aim to benefit from this groundwork in order to demonstrate the superiority of the proposed, integrated, method outlined in Figure 5.

The three minimizing optimization processes are defined as follows:

M1 Return the nearest match $k_{i}$ such that R=min(R);
M2 Solve min${∥x∥+∥x-w∥}_{1}$ for $w_{j=i}=1$; $w_{j\neq i}=0$, subject to $y=xg_{\phi }\left(K\right)$;
M3 Solve min∥x∥ subject to $y=xg_{\phi }\left(K\right)$.

To determine the triage constants α and β, we incrementally increased each value while repeatedly applying the algorithm on randomized testing and validation partitions. We also realized that β should depend on the number of channels *p* of the encoder. This is because lower-dimensional data will more effectively “mask” the rarity of the signal (i.e., leading to greater metamerism). For this reason, we repeated this analysis for p=(1,…,25) channels. While we experimented with many different relationships, the ansatz that β scales with the inverse square-root of *p* was the most successful. Consequently, we were able to determine the following relationship for the available spectral data:

(15) $$\beta =\frac{L_{\beta }}{\sqrt{p}}$$

where *L* is the target loss (of the reconstruction) determined by the spectral angle mapper [64]. In practice, $L_{\beta }=0.05$. For α, we found that this relationship also holds—albeit for a smaller target loss such that $L_{\alpha }=0.01$.

## 6. Non-Uniform Performance Dynamics

In this section, we present results from simulated data for which we compare the viability of the learned non-uniform sampling protocols against the uniform reference. We compute the mean errors and plot their distribution as a function of method and rate. Additionally, we test our hypotheses regarding the correlation between loss, signal complexity, and signal rarity.

### 6.1. Comparison to Existing Approaches

**Figure 6:**
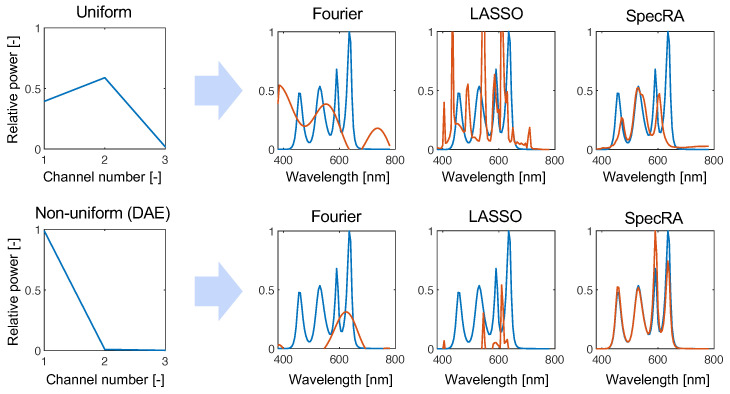
Comparing the performance of the algorithm in the “extreme” undersampled case of p=3, we see how SpecRA outperforms the other methods, mostly by avoiding overfitting. Furthermore, the performance is improved when the signal is encoded with a learned non-uniform protocol (in this case, derived from the weights learned by training a deep autoencoder network).

To obtain an idea of the differences between SpecRA and other competing approaches, we present results from the undersampled regime (p=3). Here, Figure 6, we simulated the response that a tristimulus sensor would have with uniform and non-uniform responsivity. As expected, the information being fit is too coarse for the Fourier modes to find a fit in the measured dimension. LASSO fails to find a parsimonious fit and succumbs to overfitting. Because of the adaptability of SpecRA, the fit is more balanced and the ansatz made in the low-rank space is not far from the ground truth. Furthermore, we can see that the performance increases when structure is preserved by finding a more optimal low-rank encoding.

While reconstruction with Fourier modes may be more appropriate in higher dimensional reconstruction (i.e., p>10), the advantages are only seen when the measured signal is typologically distant from the reference set of prior observations. SpecRA takes a simple yet effective approach: maximizing the available information and not overfitting.

### 6.2. Loss as a Function of Method and Rate

As we are working with spectral data, we reported the reconstruction error (loss) terms of the spectral angle mapper (SAM) defined as

(16) $$L=K\left(s,\;\hat{s}\right)=\cos^{-1}\frac{\sum_{i=1}^{n}s_{i}\;{\hat{s}}_{i}}{{\left(\sum_{i=1}^{n}s_{i}^{2}\right)}^{1/2}\;{\left(\sum_{i=1}^{n}{\hat{s}}_{i}^{2}\right)}^{1/2}}.$$

In order to compare results, we first split our library **L** into five random training, **T**, and validation **V** sets. The training sets comprised $n_{T}=300$ spectra while the validation sets comprised $n_{V}=1417$ (i.e., the remaining signals in **L** after removing the 146 theoretical sources). We then constructed our encoder using the training set to first derive **K**, then Ψ, and finally **P** used to construct the response functions **R** for $g_{\theta }$. Then, we simulated the measurement process of the spectra in the validation set by Equation (13) with ϵ(λ,p)=0 (i.e., for comparative analysis and applications where simulated data are used, e.g., rendering) for p=(1,…,25). We then reconstructed the measured spectra via the SpecRA algorithm, and computed the reconstruction loss defined above.

**Figure 7:**
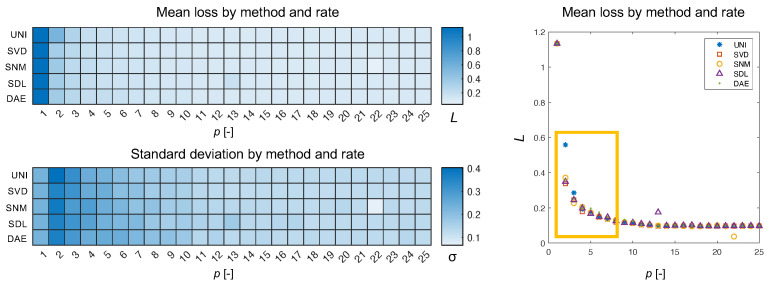
Here, we show the mean loss and standard deviation for each method as a function of the sampling rate. The methods are abbreviated by three-letter codes for visual clarity. The mean SAM loss, *L*, is displayed as a heat-map and as a Pareto plot. The difference between methods is greatest in for p<5 (boxed region).

**Figure 8:**
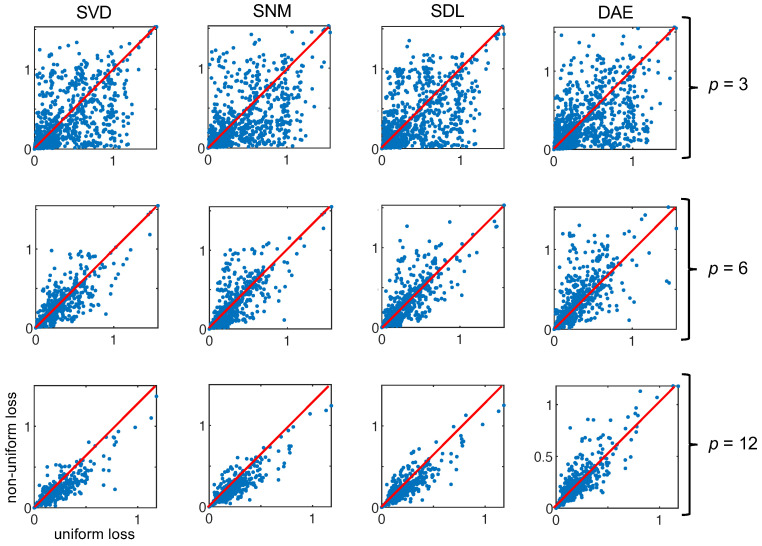
Here, we plot the uniform loss against the non-uniform loss for the four derived sampling protocols at three different encoder rates for all the spectra in our validation set ($n_{V}=1417)$. When the majority of the points fall below the y=x line (shown in red), this means that the non-uniform approach outperforms the uniform method (e.g., SVD, p=12).

What we see in Figure 7 is the type of Pareto distribution we expect to see in such experiments. As the number of channels increases, eventually a plateauing effect is observed wherein adding more channels to the encoder does not result in greater returns in minimizing loss. What is interesting is that the uniform approach is very clearly the exception resulting in a loss 150% that of the nearest non-uniform approach for an encoder with two channels. Aside from the mean, we can also plot the individual loss per reconstructed spectrum in the validation set. In Figure 8, we can see how plotting the uniform losses against the non-uniform losses directly evidences the efficacy of the underlying method. While it is clear from Figure 7, that non-uniform methods are competitive for p<4, we can see how this competitive edge is maintained for larger *p*. Even after the plateauing of the mean, we can see that the distribution of losses exhibits a bias for non-uniform methods (c.f., SVD, SNM, and SDL, for p=12). Interestingly, this effect is not observed for responses derived via the autoencoder weights which are presumed to be a generalization of the SVD modes demonstrating competitive results.

### 6.3. Correlation between Loss, Signal Complexity, and Rarity

**Figure 9:**
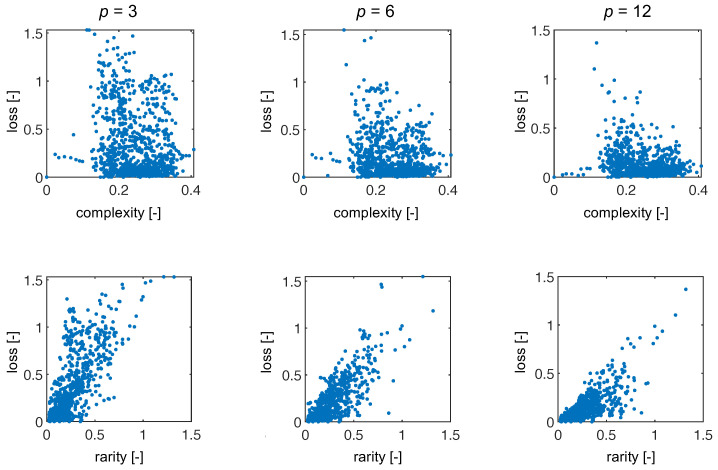
Here, we show the results for loss as a function of complexity (top row) and as a function of rarity (bottom row) for three representative encoder rates.

In addition to comparing methods, we had two core hypotheses: loss will be correlated with signal complexity and rarity. To test these hypotheses we computed the signal complexity by taking the standard deviation of the signal (as a proxy) and the signal rarity by computing the mean of the 10 most similar (according to Equation (16)) signals in the training set. Since SpecRA enforces sparsity, the number of spectra used in the reconstruction generally does not exceed 10 (or else it would suffer from overfitting). Towards this end, we compute these metrics and present the results in Figure 9. Interestingly, there is little to no correlation between the complexity of the signal and reconstruction via SpecRA while, we see a notable trend in the loss when plotted against the rarity of the signal relative to the training set (especially for lower rates). The lack of correlation with signal complexity could be a result of how the SpecRA switches between reconstruction methodologies and also explain why signal rarity does not exhibit a stronger correlation. If the spectra in the training library or predefined set, **K**, are similar to the measured spectra, SpecRA will be effective at finding either a direct match or a sparse approximation, even if the rate is small. At the same time, we see detrending as the rate increases, indicating that rarity is less important when more information is available.

The results of our preliminary analysis are promising and generally consistent with our hypotheses. One interesting question that remains unanswered here is the degree to which a signal can differ from the reference set, **K**, and still be effectively reconstructed from a sparse combination of prior observations.

## 7. Discussion and Future Integration

Formally, reconstruction, in the context of signal processing, is any process that recovers a signal from the set of points. Whether this is performed via a codebook or regression, the end goal is the same: recover information that is not readily available (i.e., via interpolation). Data-driven reconstruction methods apply some inductive bias and in the case of SpecRA, we exploit *Lex Parsimoniae* by toggling between pattern matching, $l_{1}$-minimization via a primal-dual algorithm, and linear interpolation. As more information becomes available via online repositories, tailored bases will likely outperform generic ones such as that of Fourier. As is the case with all data-driven methods, the success is still highly linked to the availability and tractability of the underlying data (which can be a challenge for hyperspectral imaging).

In this paper, we simulated response functions for uniform and non-uniform sampling methods based on exploiting regularities in the frequency domain. Applying real-world constraints on idealized mathematical models is a challenge in any discipline. In the case of visible spectral data, there is a clear effect of information saturation implying that finer resolution is perhaps not needed to capture sufficiently unique information about the spectrum. On the other hand, the relative rarity of a signal does play a role in constraining the possible loss. Towards this end, applying online learning algorithms to sift through repositories to construct interpretable low-rank reference libraries, **K**, is paramount.

Furthermore, systematically selecting filter locations via a combined process of data-driven analysis and matrix factorization can dramatically improve results, especially for low-rate encoding (i.e., p<4). A clear benefit of our method is its independence from fitted optimization parameters for a set of priors, and prior information in the form of a measured signal does improve results. Finally, our approach remains untested on real-world sensing hardware. It remains unclear what effect increasing channel errors will have on the reconstruction process. This will be investigated in future work. In applying this work to other domains, it is important to note that while many of these methods are generalizable, there will always be specific design constraints that need to be considered for any new class of sensors. In conclusion, spectral sensing spans many disciplines and its relevance in autonomous systems is becoming even more present. The systems and methods outlined in this paper provide a working template for research into the design and implementation of compact spectrometers and rendering software for a diversity of applications. While conventional spectrometry is seen as costly and data intensive, taking advantage of domain knowledge and sparse optimization can offer a valuable alternative to existing methods. We hope that this work provides a foundation for both theoretical and practical future developments.

## Data Availability

https://github.com/fwebler (accessed on 17 March 2022).
